# Author Correction: Entorhinal cortex directs learning-related changes in CA1 representations

**DOI:** 10.1038/s41586-022-05552-w

**Published:** 2022-11-15

**Authors:** Christine Grienberger, Jeffrey C. Magee

**Affiliations:** 1 Howard Hughes Medical Institute, Baylor College of Medicine, Houston, TX USA; 2 Present Address: Brandeis University, Department of Biology and Volen National Center for Complex Systems, Waltham, MA USA

**Keywords:** Hippocampus, Synaptic plasticity

Correction to: *Nature* 10.1038/s41586-022-05378-6 Published online 2 November 2022

In the version of this article initially published, the Acknowledgements section omitted mention of support from the Cullen Foundation. The section has been corrected in the HTML and PDF versions of the article.

