# Supplementary material for: Entorhinal cortex directs learning-related changes in CA1 representations
Source: Nature. 2022 Nov 2;611(7936):554–62. doi: 10.1038/s41586-022-05378-6 (PMC9668747; doi:10.1038/s41586-022-05378-6)
Supplement: Supplementary file 1 — Reporting Summary [file 41586_2022_5378_MOESM1_ESM.pdf]

Corresponding author(s): Jeffrey C. Magee

Last updated by author(s): Aug 5, 2022

Reporting Summary

Nature Portfolio wishes to improve the reproducibility of the work that we publish. This form provides structure for consistency and transparency in reporting. For further information on Nature Portfolio policies, see our Editorial Policies and the Editorial Policy Checklist.

Statistics

For all statistical analyses, confirm that the following items are present in the figure legend, table legend, main text, or Methods section.

n/a	Confirmed	
☐	☒	The exact sample size (n) for each experimental group/condition, given as a discrete number and unit of measurement
☐	☒	A statement on whether measurements were taken from distinct samples or whether the same sample was measured repeatedly
☐	☒	The statistical test(s) used AND whether they are one- or two-sided<br>*Only common tests should be described solely by name; describe more complex techniques in the Methods section.*
☐	☒	A description of all covariates tested
☐	☒	A description of any assumptions or corrections, such as tests of normality and adjustment for multiple comparisons
☐	☒	A full description of the statistical parameters including central tendency (e.g. means) or other basic estimates (e.g. regression coefficient) AND variation (e.g. standard deviation) or associated estimates of uncertainty (e.g. confidence intervals)
☐	☒	For null hypothesis testing, the test statistic (e.g. F, t, r) with confidence intervals, effect sizes, degrees of freedom and P value noted<br>*Give P values as exact values whenever suitable.*
☒	☐	For Bayesian analysis, information on the choice of priors and Markov chain Monte Carlo settings
☒	☐	For hierarchical and complex designs, identification of the appropriate level for tests and full reporting of outcomes
☐	☒	Estimates of effect sizes (e.g. Cohen's d, Pearson's r), indicating how they were calculated

Our web collection on statistics for biologists contains articles on many of the points above.

Software and code

Policy information about availability of computer code

Data collection	The Ca2+ imaging data was recorded using a National Instruments PXI system, controlled by ScanImage (R2015 and R2018, Vidrio). The behavioral data was acquired using a NI PCIe-6343 card, connected to a BNC-2090A rack-mountable break-out box, controlled by Wavesurfer (Version 0.982, open-access software, wavesurfer.janelia.org). The behavioral system was controlled via an Arduino microprocessor and custom-written Arduino and Matlab code. All histological images were acquired on the ZEISS Axio Zoom.V16 microscope equipped with ZEN 3.1 software.
Data analysis	To extract somatic Ca2+ signals of CA1 pyramidal neurons, videos were motion-corrected using SIMA (Version 1.3.2), neurons were manually drawn (using Image J version 2.0.0), and calcium traces across time were extracted using SIMA. To extract axonal EC3 Ca2+ signals, the automatic motion correction and ROI detection algorithm of the Suite2P (Version 0.6.16) analysis package was used. Further analyses of CA1 and EC3 activity were performed using custom functions written in MATLAB (Version 2019a). The modeling was performed in IGOR 8.04. Histological sections were analyzed using Image J's line plot function (Image J version 2.0.0). The code used to analyze the experimental data and perform the modeling will be available via a GitHub repository.

For manuscripts utilizing custom algorithms or software that are central to the research but not yet described in published literature, software must be made available to editors and reviewers. We strongly encourage code deposition in a community repository (e.g. GitHub). See the Nature Portfolio guidelines for submitting code & software for further information.

Data

Policy information about availability of data

All manuscripts must include a data availability statement. This statement should provide the following information, where applicable:

- Accession codes, unique identifiers, or web links for publicly available datasets
- A description of any restrictions on data availability
- For clinical datasets or third party data, please ensure that the statement adheres to our policy

The data that support the findings of this study are available from the corresponding author upon request.

Field-specific reporting

Please select the one below that is the best fit for your research. If you are not sure, read the appropriate sections before making your selection.

☒ Life sciences ☐ Behavioural & social sciences ☐ Ecological, evolutionary & environmental sciences

For a reference copy of the document with all sections, see nature.com/documents/nr-reporting-summary-flat.pdf

Life sciences study design

All studies must disclose on these points even when the disclosure is negative.

Sample size	No statistical methods were used to predetermine sample sizes. The number of mice per group was determined by previous publications on a similar behavioral task than used in our study (refs. 5, 6, 27, 54), by the expected number of active neurons or axons that can be imaged with the two-photon microscope in awake behaving mice (refs. 5, 6, 27, see also Danielson et al, Neuron, 2016). The main effects were significant with the number of mice, neurons or axons in each group, and the effects were consistent across individual mice and neurons within each group, as evident by the presentation of individual data throughout the paper.
Data exclusions	Animals were excluded from further analyses for two reasons: 1) extensive z-motion did preclude imaging of the same population of neurons throughout the recording sessions; 2) an animal did run less than 20 laps per recording session of 45-60 minutes.
Replication	We used appropriate sample sizes and indicate those throughout the manuscript. All experiments were performed independently. Our main findings are maintained across all animals, and individual data points representing individual animals are shown for most of our analyses. We do not show individual data points in those plots that contains many bins, thus making it difficult to see. All replications were successful.
Randomization	Littermate GP5.17 or littermate pOxr1- Cre mice were used and randomly assigned to each experiment. For all manipulation experiments, we compared experimental group to the appropriate control group. For the hippocampal pharmacology experiment, GP5.17 littermate mice were used and were randomly assigned to the two experimental groups (APV/SNX vs. vehicle application). For the optogenetic experiments, pOxr1-Cre litter mate mice were used and randomly assigned to the two experimental groups (viral expression of ArchT-tdtomato or tdtomato).
Blinding	Experiments and data analyses were not performed blind to the experimental conditions. This was due to the fact that the experimenter also applied the experimental drug during the recording or performed the surgeries and was thus not be able to be blinded to the experimental conditions. All analyses were performed using automatized data analyses procedures.

Reporting for specific materials, systems and methods

We require information from authors about some types of materials, experimental systems and methods used in many studies. Here, indicate whether each material, system or method listed is relevant to your study. If you are not sure if a list item applies to your research, read the appropriate section before selecting a response.

Materials & experimental systems

n/a	Involved in the study	
☒	☐	Antibodies
☒	☐	Eukaryotic cell lines
☒	☐	Palaeontology and archaeology
☐	☒	Animals and other organisms
☒	☐	Human research participants
☒	☐	Clinical data
☒	☐	Dual use research of concern

Methods

n/a	Involved in the study	
☒	☐	ChIP-seq
☒	☐	Flow cytometry
☒	☐	MRI-based neuroimaging

Animals and other organisms

Policy information about studies involving animals; ARRIVE guidelines recommended for reporting animal research

Laboratory animals	All experiments were performed in adult (older than P66 at the time of surgery) GP5.17 (n=52 mice, Janelia and Jackson Laboratories) or pOxr1-Cre (n=44 mice, Jackson Laboratories) mice of either sex. Animals were housed under an inverse 12-hour dark/12-hour light cycle (lights off at 9 am) in the Magee lab satellite facility with temperature (~21 degree Celsius) and humidity (~30-60 %) controlled.
Wild animals	this study does not involve wild animals.
Field-collected samples	this study does not involve field-collected samples.
Ethics oversight	All experiments were performed according to methods approved by the Janelia (Protocol 12-84 & 15-126) and the Baylor College of Medicine's (Protocol AN-7734) IACUC committees.

Note that full information on the approval of the study protocol must also be provided in the manuscript.
